# Clinical and Histopathological Features and Potential Pathological Mechanisms of Skin Lesions in COVID-19: Review of the Literature

**DOI:** 10.3390/dermatopathology7010002

**Published:** 2020-06-30

**Authors:** Gürkan Kaya, Aysin Kaya, Jean-Hilaire Saurat

**Affiliations:** 1 Departments of Dermatology and Clinical Pathology, University Hospital of Geneva, 1205 Geneva, Switzerland; 2 Department of Clinical Pharmacology and Toxicology, University of Geneva, 1205 Geneva, Switzerland; Aysin.Kaya@unige.ch (A.K.); Jean.Saurat@unige.ch (J.-H.S.)

**Keywords:** skin lesions, COVID-19, SARS-CoV-2, histopathology

## Abstract

In recent weeks, several reports have emerged of skin lesions with different clinical presentations in COVID-19 cases. All dermatologists should be aware of these cutaneous lesions, which may be early clinical symptoms of infection. We reviewed the literature on cutaneous manifestations in the PubMed database from December 2019 and June 2020. From the cases described as case reports or series in 57 recent articles, it appears that skin lesions (i) are highly varied, (ii) may not be related to the severity of the condition and (iii) resolve spontaneously in a few days. The frequency of these lesions in COVID-19 patients varies between 1.8% and 20.4%. The major clinical forms described were maculopapular eruptions, acral areas of erythema with vesicles or pustules (pseudochilblain), urticarial lesions, other vesicular eruptions and livedo or necrosis. The lesions were mainly localized in the trunk and extremities. The majority of patients were male, aged between 4.5 and 89 years. A minority of the patients were children presenting with acral, chilblain-like lesions, papulo-vesicular eruptions or Kawasaki disease-like pediatric inflammatory multisystem syndrome. The mean duration of the lesions was a few days, but some lasting as little as 20 min and others as long as four weeks have been reported. The mean latency time in the majority of cases was between 1 and 14 days; however, in some patients, lesions appeared 2 to 5 days before the onset of COVID-19 symptoms. The histopathological features of these lesions also vary, corresponding to the diversity of clinical manifestations. These features underline the nature of epidermal and dermal vascular lesions—and in severe cases, microvascular injury and thrombosis—associated with COVID-19, and provide important clues to their pathological mechanisms.

## 1. Introduction

In recent weeks, there have been several published reports of COVID-19 cases with skin lesions with different clinical presentations; all dermatologists should be aware of these clinical signs. In patients with COVID-19, aggravation of previous skin lesions have been noted, and allergic reactions to the different medications used for treatment may occur. However, recently, there have been reports of skin lesions that may correspond to cutaneous manifestations of SARS-CoV-2 [1].

The characteristics of skin lesions in patients with COVID-19, as described recently in case reports or case series, are summarized in the Table 1 [2,3,4,5,6,7,8,9,10,11,12,13,14,15,16,17,18,19,20,21,22,23,24,25,26,27,28,29,30,31,32,33,34,35,36,37,38,39,40,41,42,43,44,45,46,47,48,49,50,51,52,53,54,55,56,57,58]. According to these publications, in which the majority of reported patients had Fitzpatrick skin types I–III [59], the skin manifestations of COVID-19 are highly varied and nonspecific, are not necessarily related to the severity of the condition and resolve spontaneously in a few days.

We still have a lot to learn about the cutaneous manifestations associated with this disease, and there are currently more questions than answers. It is still unclear what percentage of COVID-19 patients develops cutaneous eruptions. Although 20.4% of patients (18 out of 88) in an Italian cohort developed cutaneous abnormalities [24], they were present in only 1.8% (2 out of 1099 patients) in a Chinese cohort [10].

## 2. Methods

A literature search using the following strategy was performed on the PubMed database to identify eligible articles: (COVID* or coronavirus* or SARS-CoV-2*) and (dermatol* or skin* or cutaneous*). The publication date was limited to December 2019 onward. A total of 112 papers were identified in the initial search. Abstracts and full-texts of all articles were then reviewed. Reports on different cutaneous manifestations associated with COVID-19 were included in this review (67 in total, 57 of which are listed in Table 1).

## 3. Results and Discussion

### 3.1. Clinical Manifestations

Clinical manifestations are as follows (see Table 1): generalized or localized rash (erythematous, papulovesicular, maculopapular, petechial, morbilliform, symmetrical drug-related intertriginous and flexural exanthema (SDRIFE)-like, digitate papulosquamous pityriasis rosea-like), generalized urticaria, varicelliform rash, herpes lesions (zoster), purpuric lesions (retiform purpura), livedoid lesions (livedo reticularis, livedo racemosa), acro-ischemic lesions (dry gangrene, blisters, cyanosis), erythema multiforme-like, chilblain-like lesions (COVID toes) and other lesions such as urticarial vasculitis, acute generalized exanthematous pustulosis (AGEP)-like rash, eosinophilic panniculitis, COVID mask, periorbital dyschromia, oral ulcers and COVID red half-moon nail sign. In addition, a high frequency of androgenetic alopecia has been observed in COVID-19 patients. Based on these clinical manifestations, an algorithm for the classification of COVID-19 rashes has been proposed [60]. 

In a recent Spanish study including 375 cases, five clinical patterns were described [41]: maculopapular eruptions (47%), acral areas of erythema with vesicles or pustules (pseudochilblain) (19%), urticarial lesions (19%), other vesicular eruptions (9%) and livedo or necrosis (6%).

The lesions are mainly localized in the trunk and extremities (hands and feet), sparing the face; however, lesions located on the face, neck, mouth and axillary folds have also been reported.

The majority of patients were male, aged between 4.5 and 89 years. A minority of patients were children (between 4.5 and 14 years) presenting with acral, chilblain-like lesions, papulo-vesicular eruptions on the trunk or pediatric inflammatory multisystem syndrome.

The mean duration of the lesions was a few days, but some lasting as little as 20 min and others as long as four weeks have been reported. The mean latency time (i.e., time to develop skin lesions after the appearance of the first typical symptoms of COVID-19) in the majority of cases was between 1 and 14 days; however, in some patients, lesions appeared 2 to 5 days before the onset of COVID-19 symptoms.

In the table, we have classified all reported cases according to the time of occurrence of skin lesions. In some patients, lesions appeared 2 to 5 days before the onset of COVID-19 symptoms; one such case involved an 8 year-old. The types of skin lesions in these patients were similar to those appearing up to 7 days after the onset of the first symptoms of COVID-19; this is consistent with a viral rash. In contrast, lesions appearing beyond the 7th day of ongoing COVID-19 are more vascular in nature.

COVID-19 is less frequent in children than adults (<1%), with a milder course; however, cases of pediatric inflammatory multisystem syndrome with features resembling atypical Kawasaki disease occurring several weeks after SARS-CoV-2 infection have recently been reported in children in the UK, as well as in Italy, France, Switzerland and the USA (Kawa-COVID-19) [57]. Clinical presentation includes fever, variable rash, conjunctivitis and abdominal pain, progressing to hemodynamic shock with severe myocardial involvement. Severe disease, with the need for intensive care due to myocarditis, occurred in almost half of all reported cases, with a higher risk of poor outcome for patients older than 5 years of age, and particularly for teenagers. A recent study from Italy reported a 30-fold increase in the rate of Kawasaki-like presentation during the COVID-19 pandemic among children; in many cases, nasopharyngeal swabs taken from these children were negative for COVID-19, and the association with COVID-19 infection is unclear [44].

### 3.2. Histopathological Features

Histopathological features have been reported in only a minority of patients in these articles (Table 1).

#### 3.2.1. Maculopapular Eruptions

Maculopapular eruptions show superficial perivascular dermatitis with slight lymphocytic exocytosis, swollen thrombosed vessels with neutrophils, eosinophils and nuclear debris/superficial and deep perivascular dermatitis with cuffs of lymphocytes surrounding blood vessels in a vasculitic pattern/superficial perivascular vesicular dermatitis, focal acantholytic suprabasal clefts, dyskeratotic and ballooning herpes-like keratinocytes and swollen vessels with dense lymphocyte infiltration mixed with rare eosinophils in the dermis.

#### 3.2.2. Varicella-Like Papulovasicular Exanthem

Varicella-like papulovasicular exanthems display vacuolar degeneration of the basal layer with multinucleate, hyperchromatic keratinocytes and dyskeratotic cells with no inflammatory infiltrate. According to Mahé et al., these exanthems histologically show acantholysis, intraepidermal vesicles with suprabasal clefts, prominent, “pomegranate-like” dyskeratosis and suspected viral inclusions in multinucleated cells. The authors of that report proposed the term “COVID-19-associated acantholytic rash”, rather than “varicella-like rash” [58].

#### 3.2.3. Urticarial Lesions

Urticarial lesions show perivascular infiltrate of lymphocytes, some eosinophils and upper dermal edema. Urticarial vasculitis lesions show blood extravasation and neutrophilic perivascular inflammation with prominent karyorrhexis, some macrophages with a cytoplasm full of nuclear debris and endothelial swelling, necrosis and fibrin deposition. Some urticarial lesions show slight vacuolar-type interface dermatitis with occasional necrotic keratinocytes with no eosinophils, consistent with an erythema multiforme-like pattern.

#### 3.2.4. Acral Chilblain-Like Lesions

In acral chilblain-like lesions, a diffuse dense lymphoid infiltrate of the superficial and deep dermis, as well as hypodermis, with a prevalent perivascular pattern and signs of endothelial activation, are observed.

#### 3.2.5. Purpuric and Livedoid Lesions

Purpuric and livedoid lesions show thrombogenic vasculopathy accompanied by striking and extensive deposition of C5b-9 and C4d within the microvasculature.

#### 3.2.6. Pityriasis Rosea-Like Lesions

In pityriasis rosea-like lesions, there is mild diffuse spongiosis in the epidermis and rounded spongiotic vesicles containing aggregates of lymphocytes and Langerhans cells, as well as mild papillary edema and lymphohistiocytic infiltrate in the dermis.

#### 3.2.7. Kawasaki-Like Lesions

In Kawasaki-like lesions, findings consistent with leukocytoclastic vasculitis, including necrosis of the epidermis and most of the dermis with extravasation of erythrocytes and fibrin thrombi in the capillaries, as well as infiltration of neutrophils with nuclear debris in vessel walls, and C3 and IgA deposition in a vascular pattern in direct immunofluorescence, are observed.

#### 3.2.8. Subcutaneous Lesions

Subcutaneous lesions show a predominantly lobular panniculitis with lymphocytes, histiocytes and many eosinophils, consistent with an eosinophilic panniculitis.

#### 3.2.9. Pustular Lesions

Pustular lesions show a subcorneal pustule with mild focal acanthosis and spongiosis, neutrophilic exocytosis, sparse keratinocyte necrosis and a perivascular lymphocytic infiltrate with rare neutrophils and eosinophils, consistent with acute generalized exanthematous pustulosis.

In a recent report, the postmortem histology of COVID-19 patients revealed lymphocytic endotheliitis in lung, heart, kidney, liver and small intestine, a pathological picture reminiscent of what is seen in skin lesions, suggesting that SARS-CoV-2 infection facilitates the induction of endothelial inflammation in several organs as a direct consequence of viral involvement and of host inflammatory response [61].

The histopathological features of these lesions also vary, corresponding to the diversity of clinical manifestations. In maculopapular lesions, it is sometimes quite difficult to differentiate a drug reaction from a viral infection. However, the presence of multinucleated ballooning cells suggests a cytopathic effect, lending weight to the hypothesis that the lesions are due to COVID-19. For varicella-like acantholytic lesions, Grover’s disease should be eliminated by the presence of endothelial swelling and vascular damage or extensive epidermal necrosis, and a herpes infection by immunohistochemistry and cultures. The histology of chilblain lesions are quite characteristic, with dermal edema and deep lymphocytic vasculitis, and purpuric and livedoid lesions show vascular thrombosis. In Kawasaki-like lesions and urticarial vasculitis, leukocytoclastic vasculitis is observed. Other lesions such as urticaria, pityriasis rosea-like, subcutaneous and pustular lesions do not seem to be specific. These features underline the nature of epidermal (acantholysis, multinucleated ballooning keratinocytes, dyskeratosis, necrosis) and dermal vascular (lymphocytic vasculitis, endotheliitis) lesions—and in severe cases, microvascular injury and thrombosis—associated with COVID-19, and provide important clues to their pathological mechanisms.

### 3.3. Potential Pathological Mechanisms

The pathological mechanisms of skin lesions in COVID-19 patients remain poorly understood. Cutaneous manifestations in COVID-19 may be classified into two major groups regarding their pathomechanisms [62]:

(1) clinical features similar to viral exanthems (an immune response to viral nucleotides)
(2) cutaneous eruptions secondary to systemic consequences caused by COVID-19 (especially vasculitis and thrombotic vasculopathy).

The possible actions of SARS-CoV-2 on human skin and the resulting potential dermatological manifestations can be summarized as follows [63].

SARS-CoV-2 is a single-stranded RNA virus composed of 16 nonstructural proteins (NSP 1–16) with specific roles in the replication of coronaviruses (CoVs). For example, NSP3 has the ability to block the host’s innate immune response and promote cytokine expression, while NSP5 can inhibit interferon (IFN) signaling, and NSP16 avoids MAD5 (melanoma differentiation-associated gene 5) recognition, depressing innate immunity.

Some studies have shown direct T cell viral infection by the detection SARS-like viral particles and SARS-CoV-2 RNA in T lymphocytes.

In a subset of patients, overactive immune responses may induce immunopathological conditions, or “cytokine storm” (i.e., an increase in pro-inflammatory cytokines, in particular, IL-6); these cytokines could reach the skin and stimulate dermal dendritic cells, macrophages, mast cells, lymphocytes and neutrophils, and promote eruptions such as erythema, urticarial lesions, vesicles and others (JAK inhibitors for IL-6).

Complement activation (C5b-9 and C4d) by SARS-CoV-2 spike glycoproteins has been shown in retiform purpura [18]. In pseudochilblain and purpuric lesions, an obliterative microangiopathy consisting of endothelial and intensive myointimal growth with complement activation has been observed. This mechanism, together with increased vascular permeability, could contribute to obliterative vascular lumen and hemorrhage in COVID-19 patients [64].

Aerosolized uptake of SARS-CoV-2 leads to infection of the functional receptor angiotensin-converting enzyme (ACE) type II (ACE2)-expressing target cells such as alveolar type 2 or other unknown target cells. ACE2 is present in the skin in the basal layer of the epidermis, in endothelial cells of dermal blood vessels and in eccrine adnexal tissue [65]; a direct pathogenic effect of the virus in the epidermis via ACE2, leading to acantholysis and dyskeratosis, has been proposed [58]. COVID-19-endotheliitis via ACE2 could explain the systemic impaired microcirculatory function in different vascular beds and their clinical sequelae in patients with COVID-19 [62]. SARS-CoV-2 was shown to be absent in only four studied lesional skin biopsy samples (Table 1). However, in two recent reports, its presence was confirmed by immunohistochemistry in the endothelial cells of chilblain lesions, suggesting a causal relationship between the lesions and SARS-CoV-2 [66,67]. In one of them SARS-CoV-2 particles were found in the cytoplasm of endothelial cells by electron microscopy [66]. Endothelial damage induced by the virus could be the key mechanism in the pathogenesis of COVID-19 chilblains, and perhaps also in a group of patients severely affected by COVID-19 presenting with microangiopathic damage [66].

In order for the virus to attach (spike protein, S) to ACE2, activation by TMPRSS2, a type II transmembrane serine protease, is needed. The TMPRSS2 gene is located on human chromosome 21; one of its significant features is that several androgen receptor elements (AREs) are located upstream of the transcription start site. The first intron COVID-19 could affect males more than females due to the relationship between TMPRSS2 and androgen levels; the greater prevalence of COVID-19 in males in Spain offers a potential clue to the role of androgens in increasing COVID-19 severity, due to the higher prevalence of androgenetic alopecia among patients [9].

A better understanding of the clinical, histopathological and pathogenetic aspects of COVID-19 in different organs, including skin, will help guide early diagnoses and treatment of this new and fatal human disease with intriguing cutaneous manifestations.

## Figures and Tables

**Table 1:** Clinical and histopathological characteristics of skin lesions in COVID-19 patients reported in 57 publications.

Nb & Sex	Age	Clinical Features	Localization of Skin Lesions	Time from 1st Symptoms	Mean Duration	Histopathological Features	SARS-CoV-2 RT-PCR		Ref.
							Swab	Skin Biopsy	
1 F	8 y	Papulovesicular skin eruption	Trunk	–5 days	7 days	No biopsy	positive	ND	[7]
1 M	57 y	Exanthem	Erythematous crusted papules	–2 days	10 days	Superficial perivascular vesicular dermatitis, focal acantholytic suprabasal clefts, dyskeratotic and ballooning, herpes-like keratinocytes and swollen vessels with dense lymphocyte infiltration mixed with rare eosinophils in the dermis.	positive	ND	[8]
1 F	27 y	Urticarial erythematous plaques	Face and acral invol- vement	–2 days	ND	No biopsy	positive	ND	[12]
1 M	68 y	Painful blisters	Right side of the right loin	–2 days	ND	No biopsy	positive	ND	[52]
1 F	43 y	Dusky red, nonpruritic, nonblanching dyschromia.	Periorbital skin	–2 days	A few days	No biopsy	positive	ND	[54]
1 M	50 y								
1 F	39 y	Urticarial rash	Forearms	–2 days	ND	No biopsy	positive	ND	[27]
1 M	71 y		Entire body	–a few days					
1 F	39 y	Urticarial rash	Trunk, thigh and other areas	–1 day	ND	No biopsy	ND	ND	[46]
1F	50 y	Erythematous annular and irregular wheals.	Shoulders, elbow, knee and buttocks	Onset	2 days	No biopsy	ND	ND	[47]
1F	20 y								
17 M 3 F	mean: 51 y	Sebopsoriasis (*n* = 1), facial herpes (*n* = 1), exanthem (*n* = 9), acral vasculitic eruption (*n* = 6), urticaria (*n* = 2) and varicelliform rash (*n* = 1).	Face, acral sites and entire body	Onset (*n* = 2), later (*n* = 18).	ND	Exanthem: perivascular dermatitis and vasculitis.	positive	ND	[38]
1 M	58 y	Erythematous macules arranged in a morbilliform pattern.	Legs, thighs, forearms, arms, shoulders, back, chest and abdomen	1 day	6 days	No biopsy	positive	ND	[23]
1 M	13 y	Erythemato-violaceous, rounded lesions.	Plantar surface of the first toe and dorsal surface of the second toe on the right and left feet, respectively	2 days	Around 10 days	No biopsy	not done	ND	[22]
16 M 6 F	mean: 60 y	Varicella-like papulovesicular exanthem.	Trunk and limbs	3 days	8 days	Vacuolar degeneration of the basal layer with multinucleate, hyperchromatic keratinocytes and dyskeratotic cells. Absence of inflammatory infiltrate.	positive	ND	[21]
1 F	59 y	Erythematous	Arms, trunk and lower limbs	3 days	5 days	Superficial perivascular dermatitis with slight lymphocytic exocytosis, swollen thrombosed vessels with neutrophils, eosinophils and nuclear debris.	positive	ND	[8]
1 M	16 y	Erythemato-edematous, partially eroded macules and plaques.	Dorsal aspects of the fingers	3 days	ND	Edema of the papillary dermis, superficial and deep lymphocytic infiltrate in a perivascular and strong perieccrine pattern.	positive	ND	[40]
1 F	64 y	SDRIFE-like erythematous rash.	Antecubital fossa, trunk and axillary folds	4 days	5 days	No biopsy	positive	ND	[19]
1 F	56 y	Painful ulcers with irregular margins and varying sizes in red and nonhemorrhagic background.	Hard palate	5 days	7 days	Siffuse edema with mucosal desquamation along with granulation and ulceration under the mucosa with invasion of mononuclear cells with large and glassy nuclei.	positive	ND	[55]
1 M	75 y		Anterior of the tongue	ND					
1 M	66 y	Papules with pseudovesicular aspect and superficial crusts	Trunk	6 days	10 days	Extensive epidermal necrosis with acantholysis and large multinucleated keratinocytes with ballooning degeneration in the superficial dermis, a dense perivascular lymphocytic infiltrate with some extravasated erythrocytes, neutrophils and eosinophils, dermal vessels displaying endothelial swelling with lymphocytic vasculitic alterations and endotheliitis in the absence of fibrinoid necrosis or thrombosis.	positive	negative	[43]
1 F	32 y	Urticarial rash	Trunk and limbs	6 days	ND	Perivascular infiltrate of lymphocytes, some eosinophils and upper dermal edema.	ND	ND	[4]
1 M	20 y	Diffuse, morbilliform, maculo- papular rash.	Trunk and extremities, sparing the face	6 days	ND	No biopsy	positive	ND	[14]
1 F	55 y	Small, monomorphic vesicles of 2–3 mm diameter, often excoriated at their top.	Trunk	6 days	11 days	Scantholysis, intraepidermal vesicle, suprabasal clefts, prominent, “pomegranate-like” dyskeratosis, suspected nuclear viral inclusions and multinucleated cells.	positive	ND	[58]
1 M	55 y				22 days				
1 F	89 y	Macules	Trunk and arms	7 days	8 days	Superficial and deep perivascular dermatitis with cuffs of lymphocytes surrounding blood vessels in a vasculitic pattern.	positive	ND	[8]
5 M 1 F	mean: 15 y	Red to violaceous macules and dusky, purpuric plaques.	Mid- and distal- aspects of the toes	7 days	ND	Superficial and deep perivascular and perieccrine lymphocytic infiltrate with junctional vacuolar change and lymphocytic vasculitis, with no evidence of thrombosis in the vessels.	negative	ND	[33]
1 M	81 y	Petechial lesions initially, then hemorrhagic bullae and necrotic plaques.	Fingers and toes	7 days	ND	Partial-thickness necrosis of the superficial portion of the epidermis and a mild inflammatory infiltrate in the papillary dermis composed predominantly of neutrophils, red blood cell extravasation and small vessel vasculitis with no thrombi, papillary dermal edema or extension of the infiltrate to the deep dermis.	negative	ND	[34]
15	ND	skin rash	ND	7 days	ND	No biopsy	ND	ND	[17]
1 M	67 y	Transient unilateral livedo reticularis	Right anterior thigh	7 days	19 h	No biopsy	positive	ND	[20]
1 F	47 y		Right leg	10 days	20 min				
1	ND	Sigitate papulosquamous eruption	Periumbilical area, lateral side of the trunk and thighs	8 days	7 days	Mild diffuse spongiosis in the epidermis and rounded spongiotic vesicles containing aggregates of lymphocytes and Langerhans cells, as well as mild papillary edema and lymphohistiocytic infiltrate in the dermis.	positive	negative	[30]
71 M 61 F	mean: 19.9 y	Chilblain-like (*n* = 95), erythema multiforme-like (*n* = 37)	Hands and feet	9.2 days	8.7 days	No biopsy	positive (2 in 11)	ND	[5]
1 F	84 y	Erythemato-purpuric, millimetric, coalescing macules.	Periaxillary area	11 days	ND	No biopsy	ND	ND	[15]
1 M	32 y	Retiform purpura	Buttocks	11 days	ND	Thrombogenic vasculopathy accompanied by striking and extensive deposition of C5b- 9 and C4d within the microvasculature.	positive	ND	[18]
1 F	66 y	Dusky purpuric patches	Palms and soles	11 days					
1 F	40 y	Livedo racemosa	Chest, legs and arms	ND					
1 M	ND	Erythematous and edematous plaques with a purpuric center.	Buttocks	12 days	ND	Perivascular neutrophilic inflammation and blood extravasation in the dermis with endothelial swelling, necrosis and fibrin deposition.	ND	ND	[49]
1 F	28 y	Erythematous-yellowish papules and plaques.	Both heels	13 days	ND	No biopsy	positive	ND	[3]
1 M	26 y	Erythematous, slightly edematous eruption.	Malar region, neck and ears	14 days	6 days	No biopsy	not done	ND	[13]
1 F	60 y	Distally convex, half moon-shaped red band surrounding the distal margin of the lunula.	All fingernails	14 days	Still present after 1 month	No biopsy	positive	ND	[37]
1 F	62 y	Ssymptomatic, nonitchy rash consisting of livedoid patches/livedoid macules.	Back, abdomen and face/bilateral periorbital skin, back of the nose and frontal region	14 days	24 h	No biopsy	positive	ND	[53]
1 F	60 y	Urticarial eruption	ND	16 days	ND	Slight vacuolar-type interface dermatitis with occasional necrotic keratinocytes and no eosinophils.	ND	ND	[48]
42M 32F	mean: 19.6 y	Erythematous papules (76.4%), similar to chilblains and purpuric macules (40.54%).	Hands and feet	16.15 days	ND	Lymphocytic perivascular and perieccrine infiltrate with no vascular occlusion or intravascular thrombi.	positive (1 in 11)	ND	[31]
6 M 6F	mean: 66.3 y	Itching papular exanthem.	Entire body	20.4 days	ND	Superficial perivascular inflammation with eosinophils (*n* = 1) and lichenoid pattern with eosinophils (*n* = 1).	positive	ND	[39]
1 F	50 y	Small, monomorphic vesicles of 2–3 mm diameter, often excoriated at their top.	Trunk, upper limbs, face	21 days	10 days	Acantholysis, intraepidermal vesicle, suprabasal clefts, prominent, “pomegranate-like” dyskeratosis, suspected nuclear viral inclusions and multinucleated cells.	positive	ND	[58]
1 F	70 y	Diffuse, pruritic pustular eruption.	Face, trunk and upper limbs	21 days	30 days	Subcorneal pustules with mild focal acanthosis and spongiosis, neutrophilic exocytosis, sparse keratinocyte necrosis, and a perivascular lymphocytic infiltrate with rare neutrophils and eosinophils.	ND	ND	[36]
153M 222F	mean: 49 y	Maculopapular (47%), pseudochilblain (19%), urticarial (19%), vesicular (9%) and livedo/necrosis (6%)	Extremities, Hands and feet, trunk, limbs and acral areas	before, at the same time or after	8.6 days 12.7days 6.8 days 10.4 days ND	No biopsy	positive (234 in 375)	ND	[41]
8 F 6 M	11 children mean: 14 y 3 adults mean: 29 y	Perniosis-like erythemato-violaceous papules and macules with possible bullous evolution or digital swelling or erythemato-papular targetoid lesions	Feet in eight cases, hands in four cases, both sites in two cases (elbow in one case)	ND	2–4 weeks	Acral lesions, a diffuse dense lymphoid infiltrate of the superficial and deep dermis, as well as hypodermis, with a prevalent perivascular pattern and signs of endothelial activation/Targetoid lesions of the elbows, mild superficial perivascular dermatitis.	negative (in 5)	ND	[25]
18	ND	Erythematous rash (*n* = 14), widespread urticaria (*n* = 3) or varicella-like vesicles (*n* = 1)	Trunk	ND	A few days	No biopsy	positive	ND	[24]
5	ND	Erythematous rash (*n* = 2), urticaria (*n* = 2) and herpes lesion (*n* = 1)	Face and the upper body (*n* = 4), mouth (*n* = 1)	ND	1–6 days	No biopsy	positive	ND	[11]
1F	74 y	Livedoid macules initially, then digital infarcts and ischemic necrosis	Third fingertip of the left hand	ND	ND	No biopsy	positive	ND	[32]
2 F	27 y	Red-purple papules	Dorsal side of fingers bilaterally	ND	ND	No biopsy	positive	ND	[2]
	35 y	Diffuse erythema	Subungual area of the right thumb						
1 F	19 y	erythemato-violaceous plaques	Feet and toes	ND	ND	No biopsy	ND	ND	[6]
41 M	mean: 58 y	AGA: 29 (71%) with clinically significant AGA and 16 (39%) with severe AGA	Scalp	ND	ND	No biopsy	ND	ND	[9]
2	ND	Skin rash	ND	ND	ND	No biopsy	positive	ND	[10]
1	ND	Dengue-like petechial rash.	ND	ND	ND	No biopsy	positive	ND	[16]
1 F	ND	Painful erythematous patches with residual purpura.	Trunk and hips	ND	ND	Blood extravasation and neutrophilic perivascular inflammation with prominent karyorrhexis.	positive	ND	[49]
4	ND	Erythematous-violaceous macules, sometimes more necrotic in appearance, even with blistering lesions.	Soles of the feet, finger and/or toe pads or periungual location	ND	ND	No biopsy	ND	ND	[26]
2	ND	Urticaria	ND	ND	ND	No biopsy	positive	ND	[28]
4M 3F	mean: 59 y	Cyanosis, skin bulla and dry gangrene.	Finger/Toe	ND	ND	No biopsy	ND	ND	[29]
1M	17 y	Chilblain-like lesions	Toes of both feet and heels	ND	ND	No biopsy	negative	ND	[35]
10M 7F	mean: 32 y	Ped-violaceous, edematous, rarely necrotic lesions.	Toes, feet and fingers	ND	ND	Diffuse upper dermal edema and a dense dermal (perivascular and peri-eccrine sweat gland) lymphocytic infiltrate, endothelial cell swelling and extravasated red blood cells, thrombi, fibrin and IgM deposits in vessels.	negative	negative	[42]
7M 3F	mean: 7.5 y	Polymorphic rash	ND	ND	ND	No biopsy	2 positive 8 negative	ND	[44]
8	ND	Maculopapular eruption/ exanthema/Purpuric maculo-papulo-vesicular rash/Papular erythematousexanthema/Severe macular haemorrhagic eruption.	Trunk/Trunk and limbs/ Trunk/Extremities	ND	ND	Dyskeratotic cells, ballooning multinucleated cells and sparse necrotic keratinocytes with lymphocytic satellitosis/Nests of Langerhans cells within the epidermis and diffuse telangiectatic blood vessels in the upper dermis/Perivascular spongiotic dermatitis and a dense perivascular lymphocytic infiltration eosinophilic rich around the swollen blood vessels with extravasated erythrocytes/Edematous dermis with many eosinophils, cuffs of lymphocytes around blood vessels in a lymphocytic vasculitis/Intravascular microthrombi in the small dermal vessels.	ND	ND	[45]
1M	68 y	Morbilliform rash/Purpuric lesions/Ulcerated, purpuric plaque with retiform-livedoid borders.	Trunk/Feet/Buttocks	ND	ND	Groups of apoptotic keratinocytes in the epidermis/ND/Features consistent with a thrombotic vasculopathy.	ND	ND	[46]
1 F	72 y	Erythematous and slightly edematous patches/Isolated typical target lesions.	Trunk and upper and lower limbs/Both thighs	ND	ND	Mixed perivascular and interstitial infiltrate, including lymphocytes, granulocytes, histiocytes, plasma cells and mast cells.	positive	ND	[50]
1 F	29 y	Pruriginous and painful subcutaneous nodular lesions.	Legs, thighs, forearms and left shoulder	ND	ND	Lobular panniculitis with lymphocytes, histiocytes and numerous eosinophils.	positive	ND	[51]
1 F	68 y	Painful vesicular rash.	Left side of the chest and nape of the neck	ND	ND	No biopsy	positive	ND	[52]
1 M	16 y	Painful dusky erythematous plaques.	Posterior scalp	ND	ND	Necrosis of the epidermis and most of the dermis with extravasation of erythrocytes and fibrin thrombi in the capillaries, as well as infiltration of neutrophils with nuclear debris in vessel walls.	negative	negative	[56]
8 M 8 F	mean: 10 (4.5 to 12.5)	Maculopapular rash in 13 (81%) patients.	ND	ND	ND	No biopsy	positive (11 in 16)	ND	[57]

AGA = Androgenetic alopecia. SDRIFE = Symmetrical drug-related intertriginous and flexural exanthema. ND = Not Defined.
